# Mesoproterozoic juvenile crust in microcontinents of the Central Asian Orogenic Belt: evidence from oxygen and hafnium isotopes in zircon

**DOI:** 10.1038/s41598-018-23393-4

**Published:** 2018-03-22

**Authors:** Zhen-Yu He, Reiner Klemd, Li-Li Yan, Tian-Yu Lu, Ze-Ming Zhang

**Affiliations:** 1 Key Laboratory of Deep-Earth Dynamics, Institute of Geology, Chinese Academy of Geological Sciences, 26 Baiwanzhuang Road, Beijing, 100037 China; 2 GeoZentrum Nordbayern, Friedrich-Alexander Universität Erlangen-Nürnberg, Schlossgarten 5, D-91054 Erlangen, Germany

## Abstract

We report *in situ* O and Hf isotope data of zircon grains from coeval Mesoproterozoic (ca. 1.4 Ga) igneous metamafic (amphibolite) and granitic rocks of the Chinese Central Tianshan microcontinent (CTM) in the southern Central Asian Orogenic Belt (CAOB). Zircon grains from amphibolite have mantle-like δ^18^O_VSMOW_ values of 4.7–5.6‰ and juvenile Hf isotopic compositions (*ε*_Hf_(*t*) = 8.4–15.3; *T*_DMC_ = 1.57–1.22 Ga), whereas those from granitic rocks have δ^18^O_VSMOW_ values of 5.6–7.0‰ and evolved Hf isotopic compositions (ε_Hf_(*t*) = −1.0–8.2; *T*_DMC_ = 2.09–1.62 Ga). Zircon O–Hf isotopic compositions of the metamafic and granitic rocks provide evidence for Mesoproterozoic (ca. 1.4 Ga) crustal growth and a substantial Palaeoproterozoic supracrustal component in the CTM. These findings and previous studies, reporting ca. 1.4 Ga magmatic rocks from other microcontinents of the CAOB, suggest that a large belt of Mesoproterozoic (ca. 1.4 Ga) juvenile continental crust formed in a continental terrane, fragments of which now occur over a distance of more than a thousand kilometres in the southern CAOB.

## Introduction

The Central Asian Orogenic Belt (CAOB), one of the largest Neoproterozoic to Palaeozoic accretionary orogens on Earth, has been extensively studied in order to constrain juvenile continental crustal growth during the Phanerozoic^1–4^. Microcontinents with Precambrian crystalline basement are essential components of the CAOB by constituting approximately 50% of its crust^5^. However, their geological evolution is only poorly constrained due to restricted exposure of the Precambrian rocks, which were extensively overprinted by Palaeozoic tectonic, metamorphic and magmatic events and were largely incorporated into Palaeozoic magmatic arcs^6–9^. Therefore, deciphering the crustal components of these microcontinents is critical to constrain juvenile continental growth in the CAOB. Recently, *in situ* zircon geochronology confirmed Mesoproterozoic (ca. 1.4 Ga) magmatic activity in the CAOB, which undoubtedly testifies to the occurrence of continental crust generated before amalgamation of the CAOB (Table 1)^7,10–16^. The Mesoproterozoic Era, dominated by the break-up of the Columbia supercontinent and the formation of the Rodinia supercontinent, was an important crust-forming period in many continents across the world^17–19^. But the best-preserved remnants of ca. 1.4 Ga juvenile crust occur in eastern Laurentia, SW Baltica and SW Amazonia^20–23^. In this context, Mesoproterozoic (ca. 1.4 Ga) magmatism is critical to clarify the crustal evolution of the host microcontinents in the CAOB (cf. ref.^8^).

**Table 1:** Compilation of sample locations, ages and zircon Hf isotopic compositions of the Mesoproterozoic magmatic rocks from microcontinents in the southern CAOB. The star (*) indicates SHRIMP zircon U–Pb ages; the others are LA-ICP-MS zircon U–Pb ages.

Tectonic unit	locality	Lithology	Age (Ma)	ε_Hf_(*t*)	*T*_DMC_ (Ga)	Data source
Chinese Central Tianshan	Alatage	amphibolite	1384 ± 35	8.4–15.3	1.57–1.22	This study
	Alatage	gneissic granodiorite	1437 ± 4	2.2–6.5	1.93–1.71	ref.^7^
	Alatage	gneissic granodiorite	1438 ± 5	1.5–6.2	1.97–1.73	ref.^7^
	Alatage	gneissic monzogranite	1436 ± 4	4.2–8.2	1.83–1.62	ref.^7^
	Alatage	gneissic monzogranite	1436 ± 5	0.4–5.3	2.02–1.77	ref.^7^
	Alatage	gneissic tonalites	1436 ± 5	3.1–7.7	1.88–1.65	ref.^7^
	Alatage	gneissic tonalites	1436 ± 5	−1.0–6.8	2.09–1.70	ref.^7^
	Alatage	gneissic tonalites	1436 ± 5	2.0–7.6	1.94–1.65	ref.^7^
	Weiya	granitic gneiss	1433 ± 27	0.3–7.0	2.02–1.68	ref.^7^
	Xingxingxia	granitic gneiss	1409 ± 33	−0.2–8.6	2.03–1.58	ref.^7^
Beishan	Jiujing	granitic gneiss	1408 ± 4	2.7–12.4	2.00–1.38	ref.^10^
Xilinhot block	Sonid Zuoqi	granitic gneiss	1390 ± 17	0.4–12.0	1.98–1.39	ref.^11^
	Sonid Zuoqi	granitic gneiss	1397 ± 11			ref.^12^
	Sonid Zuoqi	granitic gneiss	1371 ± 9			ref.^12^
	Sonid Zuoqi	granitic gneiss	1369 ± 11			ref.^12^
	Sonid Zuoqi	granitic gneiss	1360 ± 12			ref.^12^
Alxa block	Zongnaishan	granitic gneiss	1433 ± 17	0.1–11.9	2.19–1.44	ref.^13^
Kyrgyz North Tianshan	Makbal	eclogite	1446 ± 25*			ref.^14^
	Makbal	eclogite	1447 ± 29*			ref.^14^
	Aktyuz	rhyolite	1373 ± 5*			ref.^16^
	Aktyuz	rhyolite	1365 ± 6*			ref.^16^

**Figure 1:**
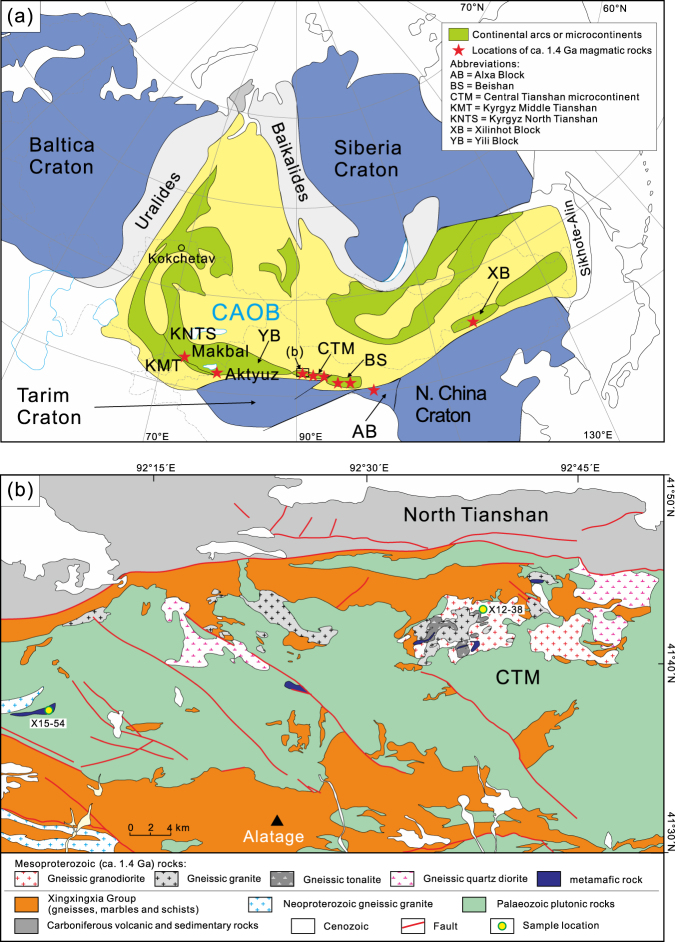
(**a**) Simplified geological map of the Central Asian Orogenic Belt. The distributions of the ca. 1.4 Ga magmatic rocks are displayed by red stars (Data sources: refs^7,10–16^). The major microcontinents in the Central Asian Orogenic Belt are also indicated, including, from west to east, the Kazakhstan, Yili, Central Tianshan, Beishan, Tuva-Mongolia and NE China microcontinental collages. (**b**) Simplified geological map of the Alatage area, showing the distribution and outline of Mesoproterozoic igneous rocks in this area. This figure was generated by Z.Y.H. using CorelDRAW 2017 (https://www.coreldraw.com/en/pages/free-download/).

However, whether ca. 1.4 Ga magmatism in the microcontinents of the CAOB was actually accompanied by significant crustal growth has largely remained speculative up to now, since the zircon Hf isotope signatures and bulk compositions of the magmatic rocks preclude derivation directly from the mantle^7,10–13,16^. However, zircon oxygen isotopic compositions are particularly useful for determining the origin of magmatic rocks since zircon in equilibrium with mantle-derived melts has a narrow δ^18^O_VSMOW_ range [5.3 ± 0.6‰ (2 SD)], which is thought to be insensitive to magmatic differentiation^24–26^. In contrast, zircon crystallized in magma from a supracrustal source has elevated δ^18^O values. Zircon oxygen isotopic compositions can also be used to track the isotopic evolution of a magmatic system through inter- or intragrain variations due to the long residence time of zircon in magma chambers^25–27^. In this study, we present *in situ* O and Hf isotope compositions of zircon grains from Mesoproterozoic magmatic metamafic and granitic rocks from the Alatage area in the Chinese Central Tianshan microcontinent (CTM) of the southern CAOB to gain insight into crustal evolution of the CTM (Fig. 1). The new data allow us to propose Mesoproterozoic (ca. 1.4 Ga) juvenile magmatism in the microcontinents of the southern CAOB.

## Results

### Field occurrence and petrography

The Chinese Tianshan is commonly subdivided into the North Tianshan, the Yili block, the CTM and the South Tianshan Accretionary Complex, and occupies major parts of the southwestern CAOB^28–30^. A Palaeozoic continental arc with Precambrian basement characterizes the CTM. The basement rocks are mainly exposed in the Xingxingxia, Weiya, Alatage and Baluntai areas and include Meso- to Neoproterozoic igneous and supracrustal rocks that were ascribed to the Xingxingxia Group^6,7,31,32^.

The Alatage metamafic rocks occur as boudins and lenses ranging from metres to tens of metres in length in Palaeozoic mylonitic granitoids and are commonly aligned with the foliation (Fig. 1b). The investigated amphibolite sample X15–54 is predominantly composed of hornblende (~60 vol.%) and plagioclase (~30 vol.%), with minor biotite, magnetite and quartz. The rock has a protolith age of 1384 ± 35 Ma (Table S1; Figs S1 and S2). The Mesoproterozoic granitic rocks in the Alatage area intruded into marble and schist of the Xingxingxia Group and were in turn intruded by Palaeozoic granitoids, occurring as sporadic outcrops (Fig. 1b). The gneissic granitoids are mylonitized to a varying extent and classify as granite, granodiorite, tonalite and quartz diorite (see details in ref.^7^). The granitic rocks were emplaced almost synchronously with the protolith of the amphibolites and have zircon crystallization ages of ca. 1438–1436 Ma^7^.

### Zircon O isotopes

**Figure 2:**
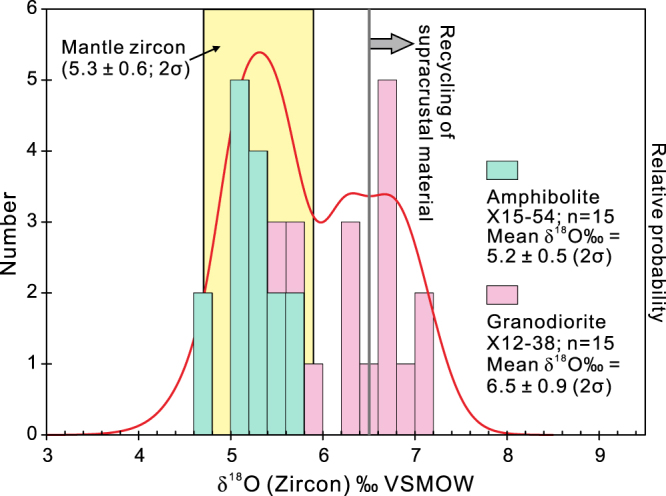
Histogram showing zircon δ^18^O_VSMOW_ values for the ca. 1.4 Ga Alatage amphibolite and gneissic granodiorite. Yellow bar represents δ^18^O_VSMOW_ of zircon in equilibrium with mantle-derived melts (5.3 ± 0.6‰, 2σ); values above 6.5‰ indicate recycling of supracrustal material^24–26^. Note the amphibolite has broadly mantle-like zircon δ^18^O values, while the zircon δ^18^O_VSMOW_ values of the gneissic granodiorite are relative high and variable.

Fifteen analyses on fifteen magmatic zircon grains from amphibolite sample X15–54 show limited intragrain O isotope variability. The δ^18^O_VSMOW_ values cluster between 4.7 and 5.6‰, with an average of 5.2 ± 0.5‰ (2 SD; Fig. 2). In addition, fifteen analyses on 15 magmatic zircon grains from gneissic granodiorite sample X12–38 revealed δ^18^O_VSMOW_ values of 5.6 to 7.0‰, with an average of 6.5 ± 0.9‰ (2 SD; Fig. 2). This intragrain δ^18^O range distinctly exceeds that of the homogeneous zircon standard Penglai (0.5‰; Table S2).

### Zircon Hf isotopic compositions

**Figure 3:**
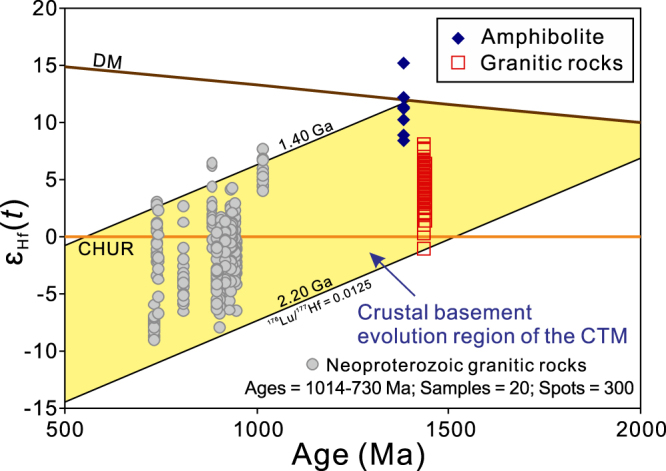
Zircon Hf isotopic evolution diagram for the ca. 1.4 Ga Alatage amphibolite and granitic rocks. Also showing the Neoproterozoic granitic rocks from the CTM (Data sources: refs^6,7,33–40^). Note that the ε_Hf_(*t*) values of the Neoproterozoic granitic rocks are typically located in the crustal basement evolution region of the CTM as defined by the Mesoproterozoic rocks. The ‘crust evolution curve’ is based on the ^176^Lu/^177^Hf value of 0.0125 for average upper continental crust^49^.

Eight Lu–Hf analyses were performed on 8 magmatic zircon grains from amphibolite sample X15–54. These analyses commonly show uniform initial ^176^Hf/^177^Hf ratios of 0.282142 to 0.282337 (Fig. 3), that correspond to *ε*_Hf_(*t*) values between 8.4 and 15.3 and crustal model ages (*T*_DMC_) of 1.57 to 1.22 Ga (Table S3). The zircon Hf data for the Alatage granitic rocks, which include gneissic granodiorite sample X12-38, were described in detail in ref.^7^. A total of 120 Hf isotopic spot analyses on zircon grains from seven samples yielded varying initial ^176^Hf/^177^Hf ratios from 0.281844 to 0.282103 and ε_Hf_(*t*) values from −1.0 to 8.2, corresponding to crustal model ages (*T*_DMC_) of 2.09 Ga to 1.62 Ga (Fig. 3).

## Discussion

### Mesoproterozoic crustal growth and nature of crustal components in the CTM

The Alatage amphibolite shows primitive zircon oxygen isotopic compositions with zircon δ^18^O_VSMOW_ values identical to those of zircon in equilibrium with mantle-derived melts (Fig. 2). The zircon Hf isotopic compositions are radiogenic and overlap the depleted mantle line (Fig. 3). The coupling of zircon O and Hf isotope compositions implies that the parental magma of the Alatage amphibolite protolith was ultimately derived from the depleted mantle, which is also in accordance with its mafic character (SiO_2_ = 50.8 wt.%). In contrast, the Alatage granitic rocks show somewhat evolved Hf isotope signatures with Palaeoproterozoic *T*_DMC_ crustal model ages (2.09 to 1.62 Ga) that are commonly older than the crystallization ages (Fig. 3; the difference is approximately 0.20 to 0.70 Ga), suggesting a mixed juvenile and recycled (metasedimentary) source or an ancient mantle-derived source (infracrustal progenitor)^24–26^. Besides, they exhibit a large range in zircon δ^18^O_VSMOW_ values from a mantle-like value to high δ^18^O_VSMOW_ values representative of recycling of sedimentary material (Fig. 2). Thus, the magma of the Alatage granitic rocks is thought to have been derived from a mixed sedimentary source and mantle melts, and thus their Hf model ages (2.09 to 1.62 Ga) may be hybrid, reflecting mixing rather than specific crust-forming events. However, a supracrustal component with an age of at least 1.62 Ga was deduced in the crust of the CTM. In summary, zircon O–Hf isotope compositions of the Alatage metamafic and granitic rocks reveal evidence for Mesoproterozoic (ca. 1.4 Ga) crustal growth and a possible Palaeoproterozoic supracrustal component in the CTM. Moreover, Neoproterozoic granitic rocks with protolith crystallization ages between ca. 1014 and 730 Ma are also abundant in the CTM^6,7,33–40^. Their ε_Hf_(*t*) values are typically located in crustal evolution region of the CTM as defined by Mesoproterozoic rocks (Fig. 3). This indicates that the Palaeoproterozoic supracrustal component and the Mesoproterozoic juvenile crust of the CTM were reworked in the Neoproterozoic.

### Mesoproterozoic (ca. 1.4 Ga) juvenile continental crust and its fragments in the southern CAOB

Mesoproterozoic magmatic events were recently identified in several microcontinents of the southern CAOB (Table 1). The present findings reveal evidence for Mesoproterozoic (ca. 1.4 Ga) crustal growth in the Alatage area of the CTM. Similar Mesoproterozoic magmatic activity at ca. 1.43–1.41 Ga involving juvenile crustal growth also occurred in the Weiya and Xingxingxia areas of the CTM with ε_Hf_(*t*) values from −0.2 to 8.6 and *T*_DMC_ crustal model ages of 2.03 to 1.58 Ga^7^. In addition, gneissic granitoids with a protolith age of 1408 ± 4 Ma were reported from the Beishan microcontinent to the east of the CTM^10^. Their radiogenic Hf isotopic compositions (*ε*_Hf_(*t*) = 2.7–12.4; *T*_DMC_ = 2.00–1.38 Ga) indicate the involvement of juvenile crust^10^. Furthermore, Mesoproterozoic (1.39–1.36 Ga) granitic rocks and associated crustal growth events were reported from the Xilinhot block in the eastern CAOB with ε_Hf_(*t*) values from 0.4 to 12.0 and *T*_DMC_ model ages of 1.98 to 1.39 Ga^11,12^. Similarly, Mesoproterozoic granitic rocks with an age of 1433 ± 17 Ma also occur in the northern Alxa block on the southeastern margin of the CAOB, and their Hf crustal model ages (2.19–1.44 Ga) suggest the involvement of juvenile material in their magma sources^13^. In particular, magmatic protolith ages of 1446 ± 25 Ma and 1447 ± 29 Ma were reported for eclogite-facies rocks from the Makbal metamorphic complex in the Kyrgyz North Tianshan, western CAOB^14,15^. Mesoproterozoic (1373–1365 Ma) volcanic rocks also occur in the Aktyuz area of the Kyrgyz North Tianshan^16^. Therefore, it is suggested that Mesoproterozoic (ca. 1.4 Ga) juvenile continental crust probably occurred along a large continental belt, now largely tectonically fragmented, ranging from the Kyrgyz North and Middle Tianshan through the Yili, Central Tianshan, Beishan and northern Alxa blocks or microcontinents in NW China to the Xilinhot block in NE China (Fig. 1a). The microcontinents are believed to have formed as part of a continental terrane, fragments of which now occur over a distance of more than a thousand kilometres in the southern CAOB. The previously unknown ca. 1.4 Ga continental crustal growth episode is a remarkable feature of the microcontinents in the CAOB and may provide important clues for the origin and evolution of the host microcontinents and thus the reconstruction of tectonic environments in the CAOB.

## Methods

### Zircon O isotopes

Zircon oxygen isotopes were measured using the Cameca 1280 SIMS at Institute of Geology and Geophysics, Chinese Academy of Sciences, Beijing. The Cs^+^ primary ion beam was accelerated at 10 kV with an intensity of ca. 2 nA. The spot diameters were ca. 10 μm. The instrumental mass fractionation factor (IMF) was corrected using the Penglai zircon standard (δ^18^O_VSMOW_ = 5.3‰)^41^. The detailed analytical procedures have been described in ref.^41^. The two standard deviation of the reproducibility of the Penglai zircon standard during the course of this study was 0.5‰ (2 SD; n = 23; Table S2), which accounts for the analytical precision. Eleven analyses of in-house zircon standard Qinghu during the course of this study yield a weighted mean of δ^18^O = 5.4 ± 0.7‰ (2 SD; Table S2), which is consistent within errors with the reported value of 5.4 ± 0.2‰^42^.

### Zircon Hf isotopic compositions

Zircon Hf isotope analyses were carried out *in situ* using a Coherent GeoLas Pro 193-nm laser ablation system combined with a Thermo Scientific Neptune Plus Multi Collector ICP-MS at the State Key Laboratory for Mineral Deposits Research, Nanjing University. Analyses were carried out with a beam diameter of 44 μm. The detailed procedure and interference correction method of ^176^Yb on ^176^Hf are described in ref.^43^. Standard Mud Tank was analysed during the course of this study and yielded a mean ^176^Hf/^177^Hf value of 0.282493 ± 44 (2 SD; n = 59; Table S3), which is consistent within error with the recommended values^44^. The measured ^176^Lu/^177^Hf ratios and the ^176^Lu decay constant of 1.867 × 10^−11^ yr^−1^ were used to calculate initial ^176^Hf/^177^Hf ratios^45^. The chondritic values of ref.^46^ were used for the calculation of the *ε*_Hf_ values. The depleted mantle Hf model age (*T*_DM_) was calculated using the analysed ^176^Lu/^177^Hf value of zircon and depleted mantle values of ref.^47^. The crustal model age (*T*_DMC_) was calculated using a ^176^Lu/^177^Hf value of 0.022 for mafic rocks and 0.009 for felsic rocks^48^.

## Electronic supplementary material

Supplementary Material
