# Supplementary material for: Mesoproterozoic juvenile crust in microcontinents of the Central Asian Orogenic Belt: evidence from oxygen and hafnium isotopes in zircon
Source: Sci Rep. 2018 Mar 22;8:5054. doi: 10.1038/s41598-018-23393-4 (PMC5864758; doi:10.1038/s41598-018-23393-4)
Supplement: Supplementary file 1 — Supplementary Material [file 41598_2018_23393_MOESM1_ESM.pdf]

Supplementary Material for

Mesoproterozoic juvenile crust in microcontinents of the Central Asian Orogenic Belt: evidence from oxygen and hafnium isotopes in zircon

Zhen-Yu He *, Reiner Klemd, Li-Li Yan, Tian-Yu Lu, and Ze-Ming Zhang

* Corresponding author. Email: ahhzy@163.com (Z.-Y. He)

Supplementary Materials include:

Supplementary text: Description of the internal structure of zircon from the Alatage amphibolite and gneissic granodiorite and zircon U–Pb age of the Alatage amphibolite (sample X15-54).

Table S1: LA-ICP-MS zircon U–Pb isotopic dating results and REE compositions (ppm) of zircon grains from the Alatage amphibolite.

Table S2: Oxygen isotope ratio of zircon grains from the Alatage amphibolite and gneissic granodiorite.

Table S3: Hf isotopic compositions of zircon grains from the Alatage amphibolite.

Internal structure of zircon. Zircon grains were extracted using standard density and magnetic separation techniques. Cathodoluminescence (CL) images of the analyzed zircon grains were obtained using an FEI NOVA NanoSEM 450 scanning electron microscope equipped with a Gatan Mono CL4 cathodoluminescence system at Institute of Geology, Chinese Academy of Geological Sciences.

Zircon grains from the Alatage amphibolite (sample X15-54) range in length from 60–100 μm. CL images indicate that all grains contain weakly luminescent cores with thickened or blurred primary zones which are surrounded by outer strongly luminescent thin rims (Fig. S1). The rims commonly transgress the core domains and in places penetrate into the core along cracks, which were probably formed from fluid-present recrystallization (cf. ref. 1).

Zircon grains separated from the Alatage gneissic granodiorite samples X12-38 range in length from 200 to 300 μm. They display similar internal textures that are characterized by magmatic oscillatory zoned cores surrounded by CL-dark mantles. An outer thin light rim

with a few microns thickness is commonly observed (Fig. S1). These zircon grains are interpreted to be magmatic zircon probably affected by partial recrystallization during later mylonitization and amphibolite-facies metamorphism (cf. ref. 1).

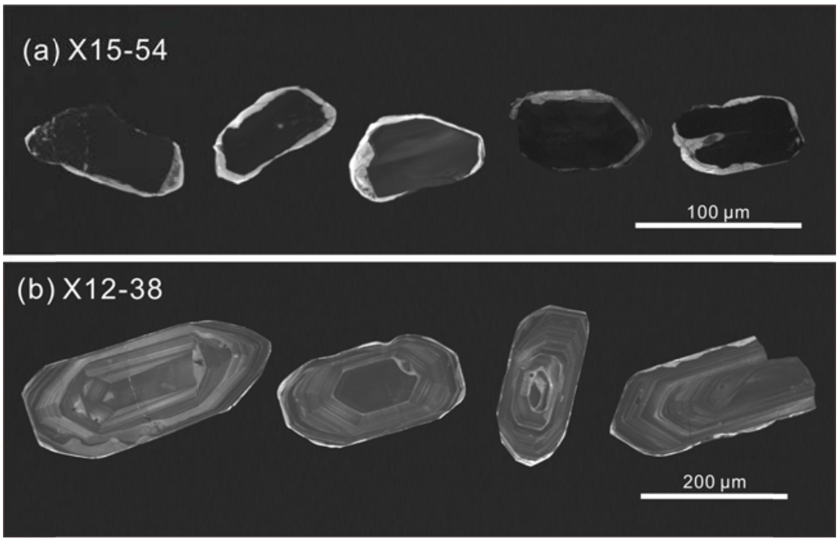

Figure S1. CL images of zircon grains from the Alatage amphibolite (a) and gneissic granodiorite (b).

LA-ICP-MS zircon U–Pb dating. Zircon U–Pb isotope and trace element analyses were carried out synchronously using an Agilent 7500a ICP-MS equipped with a 193-nm GeoLas 2005 laser ablation system at the State Key Laboratory of GPMR, China University of Geosciences, Wuhan. Analyses were carried out with a beam diameter of 32 μm. Detailed instrument conditions, analytical and data reduction procedures are given in ref. 2.

Twenty-two analyses on 28 zircon cores from amphibolite sample X15-54 yielded variable $^{206}Pb/^{238}U$ ages (925–1462 Ma) and consistent $^{207}Pb/^{206}Pb$ ages within errors (1169–1524 Ma) (Fig. S2a; Table S1), implying incomplete re-setting of the U–Pb isotopic system. Their Th/U ratios are high (0.50–2.00) and chondrite-normalized REE patterns are generally characterized by an enrichment in HREE, with positive Ce anomalies and negative Eu anomalies, all of which typical for magmatic zircon (Fig. S2b). Therefore, we interpret the upper intercept age (1384±35 Ma) as the crystallization age of the amphibolite protolith (Fig. S2a).

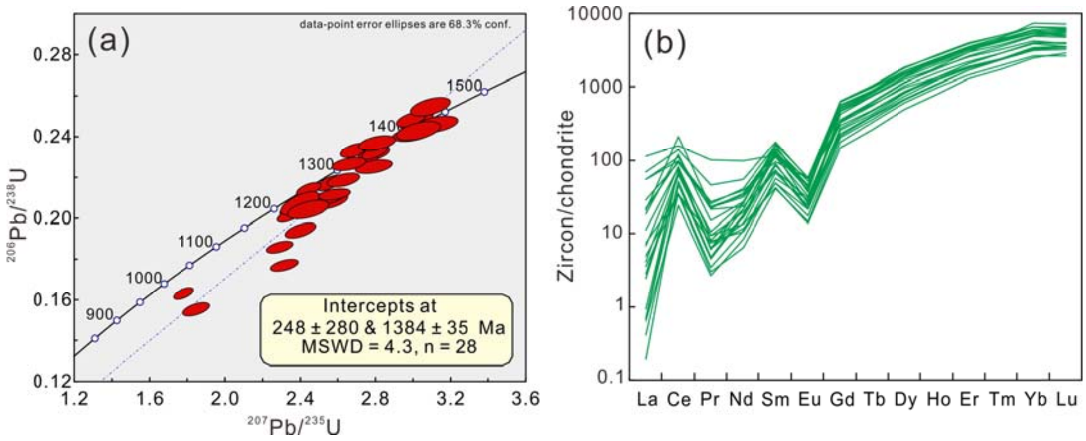

Figure S2. (a) U–Pb concordia diagram and (b) chondrite-normalized REE patterns of zircon grains from the Alatage amphibolite (sample X15-54). The chondrite values are from ref. 3.

References

1. Vavra, G., Schmid, R. & Gebauer, D. Internal morphology, habit and U-Th-Pb microanalysis of amphibolite-to-granulite facies zircons: geochronology of the Ivrea Zone (Southern Alps). *Contrib Mineral Petrol* **134**, 380-404 (1999).
2. Liu, Y. *et al.* Continental and oceanic crust recycling-induced melt–peridotite interactions in the Trans-North China Orogen: U–Pb dating, Hf isotopes and trace elements in zircons from mantle xenoliths. *Journal of Petrology* **51**, 537-571 (2010).
3. Taylor, S. R. & McLennan, S. M. The continental crust: its composition and evolution. (1985).

Table S1. LA-ICP-MS zircon U–Pb isotopic dating results and REE compositions (ppm) of zircon grains from the Alatage amphibolite

Analysis	Th (ppm)	U (ppm)	Th/U	Isotope ratios								Ages (Ma)								La	Ce	Pr	Nd	Sm	Eu	Gd	Tb	Dy	Ho	Er	Tm	Yb	Lu
				$^{207}Pb/^{206}Pb$	± 1σ	$^{207}Pb/^{235}U$	± 1σ	$^{206}Pb/^{238}U$	± 1σ	$^{208}Pb/^{232}Th$	± 1σ	$^{207}Pb/^{206}Pb$	± 1σ	$^{207}Pb/^{235}U$	± 1σ	$^{206}Pb/^{238}U$	± 1σ	$^{208}Pb/^{232}Th$	± 1σ														
X15-54-01	723	556	1.30	0.0889	0.0018	2.2828	0.0464	0.1851	0.0020	0.0571	0.0015	1411	38	1207	14	1095	11	1123	28	9.8	61.2	7.62	49.6	25.1	4.92	36.9	6.74	71.1	25.7	137	30.4	345	72.7
X15-54-02	1600	1107	1.45	0.0829	0.0015	2.6836	0.0504	0.2330	0.0023	0.0570	0.0016	1278	35	1324	14	1350	12	1120	31	24.5	130	6.08	38.1	39.9	4.85	192	57.7	673	217	921	170	1540	217
X15-54-04	1280	1053	1.22	0.0868	0.0016	2.7871	0.0553	0.2314	0.0024	0.0680	0.0018	1367	35	1352	15	1342	13	1330	34	2.45	76.7	0.85	9.71	19.0	2.34	107	35.0	429	142	627	121	1148	171
X15-54-05	602	580	1.04	0.0880	0.0015	3.0170	0.0581	0.2476	0.0031	0.0721	0.0016	1383	32	1412	15	1426	16	1407	30	0.07	43.1	0.61	11.7	26.5	3.73	143	45.2	541	177	755	140	1268	178
X15-54-06	491	446	1.10	0.0893	0.0017	2.3952	0.0534	0.1933	0.0025	0.0594	0.0013	1411	37	1241	16	1139	13	1167	24	1.78	32.3	1.26	9.34	9.49	1.18	44.8	14.8	181	66.0	310	60.2	587	105
X15-54-07	870	842	1.03	0.0890	0.0018	2.5640	0.0567	0.2078	0.0025	0.0690	0.0020	1406	38	1290	16	1217	14	1348	37	1.49	64.4	1.42	18.5	32.7	4.45	168	50.4	606	199	852	162	1537	224
X15-54-08	830	809	1.03	0.0947	0.0020	2.3084	0.0484	0.1761	0.0020	0.0574	0.0011	1524	39	1215	15	1046	11	1127	21	6.78	43.8	4.90	28.1	10.5	2.64	20.6	5.11	69.7	28.1	156	35.9	403	81.2
X15-54-09	1352	987	1.37	0.0868	0.0014	2.7387	0.0446	0.2273	0.0019	0.0648	0.0010	1367	31	1339	12	1320	10	1269	19	4.10	74.1	1.27	13.8	16.0	2.61	97.3	31.5	374	128	536	100	929	131
X15-54-11	629	831	0.76	0.0847	0.0014	2.5493	0.0467	0.2167	0.0025	0.0666	0.0011	1309	30	1286	13	1264	13	1303	21		64.2	0.87	11.6	20.5	1.86	125	42.1	514	179	794	147	1416	211
X15-54-12	379	751	0.50	0.0821	0.0014	2.4377	0.0433	0.2137	0.0022	0.0693	0.0011	1250	32	1254	13	1249	12	1354	22	2.67	31.3	1.01	8.09	11.0	1.47	61.8	20.1	241	87.7	402	81.8	822	129
X15-54-13	721	777	0.93	0.0866	0.0015	2.9865	0.0525	0.2484	0.0025	0.0747	0.0013	1354	32	1404	13	1430	13	1456	24	0.25	48.4	0.64	12.6	26.1	3.35	151	46.8	555	186	794	148	1370	191
X15-54-14	2907	1660	1.75	0.0855	0.0019	1.8393	0.0477	0.1543	0.0022	0.0328	0.0010	1328	43	1060	17	925	12	652	20	75.7	146	10.6	48.1	26.6	3.36	90.0	26.1	292	101.2	472	100.0	1079	175
X15-54-15	958	831	1.15	0.0834	0.0017	2.3603	0.0506	0.2043	0.0026	0.0678	0.0015	1280	41	1231	15	1198	14	1325	28	0.88	59.1	1.09	14.9	26.9	2.21	136	42.3	469	149	613	111	1007	141
X15-54-16	3992	2227	1.79	0.0838	0.0014	2.3357	0.0425	0.2008	0.0022	0.0479	0.0009	1289	31	1223	13	1180	12	946	18	40.8	149	13.6	67.6	26.3	4.82	89.6	26.9	314	110	493	98.5	953	140
X15-54-17	2341	1379	1.70	0.0788	0.0014	1.7721	0.0347	0.1622	0.0019	0.0323	0.0007	1169	68	1035	13	969	11	642	14	19.7	92.2	3.42	28.6	36.1	3.13	185	54.4	612	198	822	153	1403	209
X15-54-18	3870	1934	2.00	0.0867	0.0013	2.7933	0.0468	0.2323	0.0025	0.0676	0.0012	1354	29	1354	13	1347	13	1323	22	7.65	90.6	3.59	27.1	21.5	4.74	102	33.9	418	151	708	157	1732	264
X15-54-19	747	693	1.08	0.0896	0.0021	2.9914	0.0693	0.2409	0.0024	0.0687	0.0013	1417	46	1405	18	1391	12	1343	25	0.26	70.8	0.85	17.7	37.7	4.97	188	56.1	661	222	924	170	1535	229
X15-54-20	1105	937	1.18	0.0867	0.0019	2.6241	0.0570	0.2182	0.0023	0.0652	0.0013	1355	37	1307	16	1272	12	1277	24	7.85	92.2	2.93	24.3	30.5	3.32	164	50.1	577	189	784	143	1294	201
X15-54-21	1620	913	1.78	0.0875	0.0020	2.5437	0.0592	0.2096	0.0023	0.0582	0.0013	1372	44	1285	17	1227	12	1143	24	7.47	195	3.04	21.9	24.9	2.74	108	32.4	354	110	457	81.9	748	117
X15-54-22	1080	889	1.21	0.0894	0.0021	2.7876	0.0655	0.2249	0.0023	0.0605	0.0013	1414	45	1352	18	1308	12	1188	25	0.34	67.7	0.82	15.1	29.4	3.85	156	48.6	566	188	793	144	1286	190
X15-54-23	928	779	1.19	0.0847	0.0020	2.6555	0.0587	0.2262	0.0022	0.0593	0.0016	1309	46	1316	16	1315	11	1164	31	10.2	86.7	3.13	23.2	28.0	3.95	139	43.3	490	166	702	130	1196	187
X15-54-24	318	410	0.78	0.0856	0.0021	2.8055	0.0648	0.2366	0.0024	0.0653	0.0012	1329	48	1357	17	1369	13	1279	22	0.23	22.7	0.36	5.94	12.9	2.42	69.1	22.9	279	99.9	435	82.9	797	127
X15-54-25	265	352	0.75	0.0913	0.0023	3.1202	0.0770	0.2459	0.0027	0.0744	0.0015	1454	48	1438	19	1417	14	1451	28	1.30	32.2	0.41	4.54	9.28	1.23	52.3	18.6	225	78.7	348	66.7	630	96.6
X15-54-26	5570	4182	1.33	0.0832	0.0020	2.3932	0.0684	0.2067	0.0038	0.0551	0.0018	1273	47	1241	20	1211	20	1085	34	19.8	102	2.92	16.5	16.6	1.84	77.0	24.0	296	109	503	98.7	945	146
X15-54-27	232	298	0.78	0.0864	0.0027	2.4360	0.0734	0.2037	0.0031	0.0587	0.0017	1346	62	1253	22	1195	16	1153	32	5.02	32.8	5.88	35.1	16.4	2.79	21.1	2.86	27.0	9.25	46.7	11.0	141	34.2
X15-54-28	724	796	0.91	0.0874	0.0020	3.0894	0.0711	0.2546	0.0030	0.0734	0.0014	1370	44	1430	18	1462	16	1432	26	1.02	59.1	1.06	7.31	10.9	1.85	64.9	21.7	250	90.1	402	79.1	752	118
X15-54-29	431	427	1.01	0.0899	0.0022	3.0252	0.0777	0.2427	0.0031	0.0669	0.0017	1433	46	1414	20	1401	16	1309	32	0.15	32.8	0.47	7.67	15.0	2.02	81.9	25.9	305	104	445	83.0	777	118
X15-54-30	1098	1005	1.09	0.0882	0.0019	2.5758	0.0567	0.2111	0.0019	0.0626	0.0011	1387	41	1294	16	1235	10	1227	22	6.62	112	2.03	19.1	35.3	3.58	183	57.5	664	224	941	172	1555	231

Table S2. Oxygen isotope ratio of zircon grains from the Alatage amphibolite and gneissic granodiorite

Analysis	$\delta^{18}O$ (‰, VSMOW)	2σ (‰)	Analysis	$\delta^{18}O$ (‰, VSMOW)	2σ (‰)
X15-54@8	4.68	0.46	X12-38@8	5.58	0.25
X15-54@12	4.76	0.31	X12-38@1	5.69	0.35
X15-54@9	5.02	0.20	X12-38@10	5.97	0.24
X15-54@13	5.07	0.36	X12-38@5	6.22	0.24
X15-54@15	5.12	0.25	X12-38@13	6.24	0.20
X15-54@14	5.16	0.35	X12-38@15	6.25	0.31
X15-54@5	5.17	0.24	X12-38@4	6.42	0.30
X15-54@1	5.22	0.36	X12-38@6	6.68	0.33
X15-54@7	5.24	0.42	X12-38@7	6.69	0.36
X15-54@3	5.33	0.24	X12-38@2	6.72	0.24
X15-54@4	5.33	0.36	X12-38@14	6.74	0.29
X15-54@10	5.43	0.30	X12-38@11	6.78	0.47
X15-54@11	5.49	0.24	X12-38@3	6.93	0.40
X15-54@6	5.61	0.35	X12-38@9	7.03	0.30
X15-54@2	5.61	0.22	X12-38@12	7.04	0.28
**2SD**	**0.54**		**2SD**	**0.92**	
**Mean**	**5.22**		**Mean**	**6.47**	
Penglai1@4	4.77	0.27	Qinghu@6	4.76	0.19
Penglai2@15	4.86	0.21	Qinghu@9	4.94	0.30
Penglai2@1	4.95	0.30	Qinghu@2	5.18	0.34
Penglai1@2	5.03	0.32	Qinghu@11	5.19	0.27
Penglai2@2	5.04	0.27	Qinghu@7	5.35	0.30
Penglai2@7	5.06	0.27	Qinghu@5	5.36	0.41
Penglai2@5	5.12	0.25	Qinghu@10	5.44	0.30
Penglai1@6	5.14	0.21	Qinghu@1	5.45	0.50
Penglai2@10	5.15	0.35	Qinghu@3	5.51	0.45
Penglai2@16	5.20	0.41	Qinghu@8	5.93	0.24
Penglai2@9	5.21	0.49	Qinghu@4	5.96	0.34
Penglai1@1	5.22	0.37	**2SD**	**0.72**	
Penglai1@3	5.23	0.32	**Mean**	**5.37**	
Penglai1@8	5.28	0.24			
Penglai1@5	5.29	0.36			
Penglai2@3	5.31	0.29			
Penglai2@6	5.43	0.32			
Penglai2@14	5.45	0.44			
Penglai2@13	5.49	0.29			
Penglai1@7	5.52	0.33			
Penglai2@8	5.57	0.32			
Penglai2@4	5.65	0.31			
Penglai1@9	5.76	0.43			
**2SD**	**0.50**				

Note: $\delta^{18}O$ (‰) = $((^{18}O/^{16}O_{Sample})/(^{18}O/^{16}O_{VSMOW})-1)\times1000$. The standard deviation (2SD) of each sample are listed in bold.

Table S3. Hf isotopic compositions of zircon grains from the Alatage amphibolite

Analysis	Age (Ma)	$^{176}Hf/^{177}Hf$	2σ	$^{176}Lu/^{177}Hf$	$^{176}Yb/^{177}Hf$	$(^{176}Hf/^{177}Hf)_i$	$\varepsilon_{Hf}(t)$	2σ	T_{DM} (Ga)	T_{DMC} (Ga)
X15-54-01	1384	0.282191	0.000029	0.001292	0.036296	0.282157	8.9	1.0	1.51	1.54
X15-54-04	1384	0.282279	0.000041	0.003268	0.096595	0.282193	10.2	1.4	1.46	1.48
X15-54-06	1384	0.282379	0.000049	0.001578	0.043550	0.282337	15.3	1.7	1.25	1.22
X15-54-07	1384	0.282361	0.000030	0.000962	0.025380	0.282335	15.3	1.1	1.26	1.22
X15-54-08	1384	0.282271	0.000043	0.001916	0.055836	0.282221	11.2	1.5	1.42	1.43
X15-54-09	1384	0.282189	0.000034	0.001771	0.052615	0.282142	8.4	1.2	1.53	1.57
X15-54-13	1384	0.282299	0.000041	0.002840	0.081545	0.282224	11.3	1.4	1.41	1.42
X15-54-14	1384	0.282257	0.000034	0.000309	0.005139	0.282249	12.2	1.2	1.38	1.38
Standard Mud Tank										
MT-1.xls		0.282513	0.000016	0.000025	0.000748					
MT-10.xls		0.282461	0.000013	0.000042	0.001251					
MT-11.xls		0.282451	0.000013	0.000021	0.000619					
MT-12.xls		0.282440	0.000011	0.000017	0.000521					
MT-13.xls		0.282475	0.000015	0.000030	0.000915					
MT-14.xls		0.282452	0.000014	0.000019	0.000586					
MT-15.xls		0.282471	0.000014	0.000030	0.000933					
MT-16.xls		0.282420	0.000013	0.000019	0.000566					
MT-17.xls		0.282467	0.000013	0.000019	0.000574					
MT-18.xls		0.282463	0.000014	0.000030	0.000875					
MT-19.xls		0.282464	0.000014	0.000032	0.000930					
MT-2.xls		0.282496	0.000014	0.000028	0.000818					
MT-20.xls		0.282495	0.000014	0.000030	0.000879					
MT-21.xls		0.282482	0.000012	0.000019	0.000554					
MT-25.xls		0.282505	0.000013	0.000020	0.000582					
MT-26.xls		0.282499	0.000013	0.000021	0.000600					
MT-27.xls		0.282488	0.000013	0.000032	0.000920					
MT-28.xls		0.282493	0.000013	0.000036	0.001060					
MT-29.xls		0.282481	0.000014	0.000027	0.000767					
MT-3.xls		0.282498	0.000015	0.000038	0.001123					
MT-30.xls		0.282498	0.000013	0.000060	0.001802					
MT-31.xls		0.282540	0.000042	0.000060	0.001740					
MT-32.xls		0.282487	0.000017	0.000028	0.000809					
MT-35.xls		0.282500	0.000014	0.000027	0.000802					
MT-36.xls		0.282463	0.000014	0.000067	0.002043					
MT-37.xls		0.282483	0.000014	0.000028	0.000826					
MT-4.xls		0.282500	0.000014	0.000023	0.000689					
MT-45.xls		0.282489	0.000014	0.000039	0.001148					
MT-46.xls		0.282502	0.000014	0.000067	0.002074					
MT-47.xls		0.282520	0.000014	0.000062	0.001891					
MT-48.xls		0.282498	0.000015	0.000039	0.001152					
MT-49.xls		0.282525	0.000013	0.000057	0.001723					
MT-5.xls		0.282508	0.000014	0.000021	0.000619					
MT-50.xls		0.282490	0.000015	0.000022	0.000648					
MT-51.xls		0.282517	0.000013	0.000069	0.002114					
MT-52.xls		0.282508	0.000014	0.000020	0.000586					
MT-53.xls		0.282504	0.000013	0.000025	0.000710					
MT-54.xls		0.282512	0.000014	0.000020	0.000588					
MT-55.xls		0.282499	0.000016	0.000021	0.000619					
MT-56.xls		0.282495	0.000014	0.000024	0.000706					
MT-57.xls		0.282506	0.000019	0.000020	0.000560					
MT-58.xls		0.282505	0.000017	0.000022	0.000605					
MT-59.xls		0.282492	0.000017	0.000030	0.000827					
MT-6.xls		0.282492	0.000013	0.000018	0.000526					
MT-60.xls		0.282508	0.000015	0.000012	0.000336					
MT-61.xls		0.282519	0.000015	0.000016	0.000441					
MT-62.xls		0.282506	0.000012	0.000018	0.000505					
MT-63.xls		0.282503	0.000013	0.000016	0.000451					
MT-64.xls		0.282503	0.000014	0.000014	0.000403					

MT-65.xls	0.282498	0.000014	0.000014	0.000399
MT-66.xls	0.282507	0.000013	0.000012	0.000354
MT-67.xls	0.282497	0.000013	0.000012	0.000348
MT-68.xls	0.282492	0.000013	0.000014	0.000392
MT-69.xls	0.282494	0.000012	0.000016	0.000464
MT-7.xls	0.282502	0.000012	0.000020	0.000595
MT-70.xls	0.282503	0.000013	0.000015	0.000427
MT-71.xls	0.282527	0.000013	0.000013	0.000384
MT-8.xls	0.282466	0.000015	0.000027	0.000797
MT-9.xls	0.282487	0.000014	0.000035	0.001045
	AVERAGE	2SD		
	0.282493	0.000044		
